# Point and trend accuracy of continuous glucose monitoring using intravenous microdialysis in critically ill patients

**DOI:** 10.1186/cc14452

**Published:** 2015-03-16

**Authors:** JH Leopold, RT Van Hooijdonk, M Boshuizen, T Winters, LD Bos, A Abu-Hanna, MJ Schultz

**Affiliations:** 1 Academic Medical Center, Amsterdam, the Netherlands

## Introduction

Insulin infusion in critically ill patients mandates frequent measurements of the blood glucose level [1]. Microdialysis is a well-established technology that offers the opportunity to sample blood analytes with high accuracy, without the need for drawing blood samples [2,3]. We aimed to determine point and trend accuracy of microdialysis-based continuous glucose monitoring (CGM) (EIRUS®; Maquet Critical Care AB, Solna, Sweden).

## Methods

Patients with an expected stay in the ICU of >48 hours needing an arterial catheter and a central venous catheter (CVC) were eligible. For a maximum of 3 days, during 8 hours per day, 125 μl blood was drawn from the arterial line every 15 minutes. Point accuracy was expressed using Clarke error grids, Bland-Altman plots and glucose prediction error analysis [4,5]. Trend accuracy was expressed using continuous glucose error grid analysis [6].

## Results

Three-hundred and fifty-four paired samples were obtained from seven patients (66 (59 to 79) years old, APACHE II score 23 (20 to 28), 51 (19 to 77) samples per patient). Point accuracy: 91% of paired values were in zone A, with the remaining 9% of the values in zone B in the Clarke error grid. In the Bland-Altman, bias was 5.4 mg/dl with an upper limit of agreement of 32.5 mg/dl and a lower level of agreement of -21.8 mg/dl. Glucose prediction error analysis showed that 91% of the values ≥75 mg/dl within 20% of the values measured by the blood gas analyzer were within range. Trend accuracy: in the rate error grid of the continuous glucose error grid analysis, 96% of the paired values were in zone A, 3.7% were in zone B and 0.3% were in zone C.

## Conclusion

Point and trend accuracy of the tested microdialysis-based CGM are good in critically ill patients.

## Acknowledgement

Maquet Critical Care AB provided two CGM systems and disposables for the duration of the study, but had no influence on study design or study reporting.

## References

[B1] Schultz MJ Crit Care 2010 14 223 10.1186/cc8966 20550725 PMC2911685

[B2] Schierenbeck F Diabetes Technol Ther 2015 15 26 31 23137021 10.1089/dia.2012.0169

[B3] Blixt C Crit Care 2013 17 R87 10.1186/cc12713 23663747 PMC3707045

[B4] Krouwer JS J Diabetes Sci Technol 2010 4 75 83 10.1177/193229681000400110 20167170 PMC2825627

[B5] Clarke WL Diabetes Care 1987 10 622 8 10.2337/diacare.10.5.622 3677983

[B6] Kovatchev Diabetes Care 2004 27 1922 8 10.2337/diacare.27.8.1922 15277418

